# Emerging Roles for Phase Separation of RNA-Binding Proteins in Cellular Pathology of ALS

**DOI:** 10.3389/fcell.2022.840256

**Published:** 2022-02-17

**Authors:** Katarina Milicevic, Branislava Rankovic, Pavle R. Andjus, Danijela Bataveljic, Dragomir Milovanovic

**Affiliations:** ^1^ Center for Laser Microscopy, Faculty of Biology, Institute of Physiology and Biochemistry “Ivan Djaja”, University of Belgrade, Belgrade, Serbia; ^2^ Laboratory of Molecular Neuroscience, German Center for Neurodegenerative Diseases (DZNE), Berlin, Germany

**Keywords:** liquid-liquid phase separation, FUS, TDP-43, RNP aggregates, stress granule, neurons, amyotrophic lateral sclerosis

## Abstract

Liquid-liquid phase separation (LLPS) is emerging as a major principle for the mesoscale organization of proteins, RNAs, and membrane-bound organelles into biomolecular condensates. These condensates allow for rapid cellular responses to changes in metabolic activities and signaling. Nowhere is this regulation more important than in neurons and glia, where cellular physiology occurs simultaneously on a range of time- and length-scales. In a number of neurodegenerative diseases, such as Amyotrophic Lateral Sclerosis (ALS), misregulation of biomolecular condensates leads to the formation of insoluble aggregates—a pathological hallmark of both sporadic and familial ALS. Here, we summarize how the emerging knowledge about the LLPS of ALS-related proteins corroborates with their aggregation. Understanding the mechanisms that lead to protein aggregation in ALS and how cells respond to these aggregates promises to open new directions for drug development.

## Introduction

Amyotrophic Lateral Sclerosis (ALS) is a fatal neurodegenerative disease caused by the selective death of motor neurons in the spinal cord and brain. The onset of symptoms in ALS patients is observed at the age between 51 and 66 years, usually displayed as muscle weakness and impaired ability to control swallowing and speaking (Brown and Al-Chalabi, 2017; Longinetti and Fang, 2019). Most of the ALS cases are sporadic (sALS), whereas approximately 10% of cases represent a familial form (fALS) (Kim et al., 2020). The first identified mutation linked to ALS was in the gene encoding cytoplasmic enzyme superoxide dismutase 1 (SOD1) (Rosen et al., 1993). Currently, over 40 additional genes related to ALS have been discovered with the most common genetic mutation in the chromosome nine open reading frame 72 (*C9orf72*) gene observed in ∼40% of fALS patients (Taylor et al., 2016). A number of ALS-related genes encode RNA-binding proteins (RBPs) including TAR DNA-binding protein 43 (TDP-43), fused in sarcoma (FUS), heterogeneous nuclear ribonucleoprotein A1 (hnRNPA1) and T-cell restricted intracellular antigen-1 (TIA-1) (Zhao et al., 2018; Kim et al., 2021). RBPs play an important role in the regulation of RNA metabolism (Nussbacher et al., 2015, 2019; Xue et al., 2020) and many of them are prone to undergo liquid-liquid phase separation (LLPS) and form fluid condensates (Lin et al., 2015; Mittag and Parker, 2018). Notably, RBPs display tendency to aggregate and their presence is detected in the intracellular cytoplasmic aggregates, the key element in degenerating motor neurons of patients with ALS (Blokhuis et al., 2013). However, exact mechanisms driving the protein aggregation are still unknown.

**FIGURE 1:**
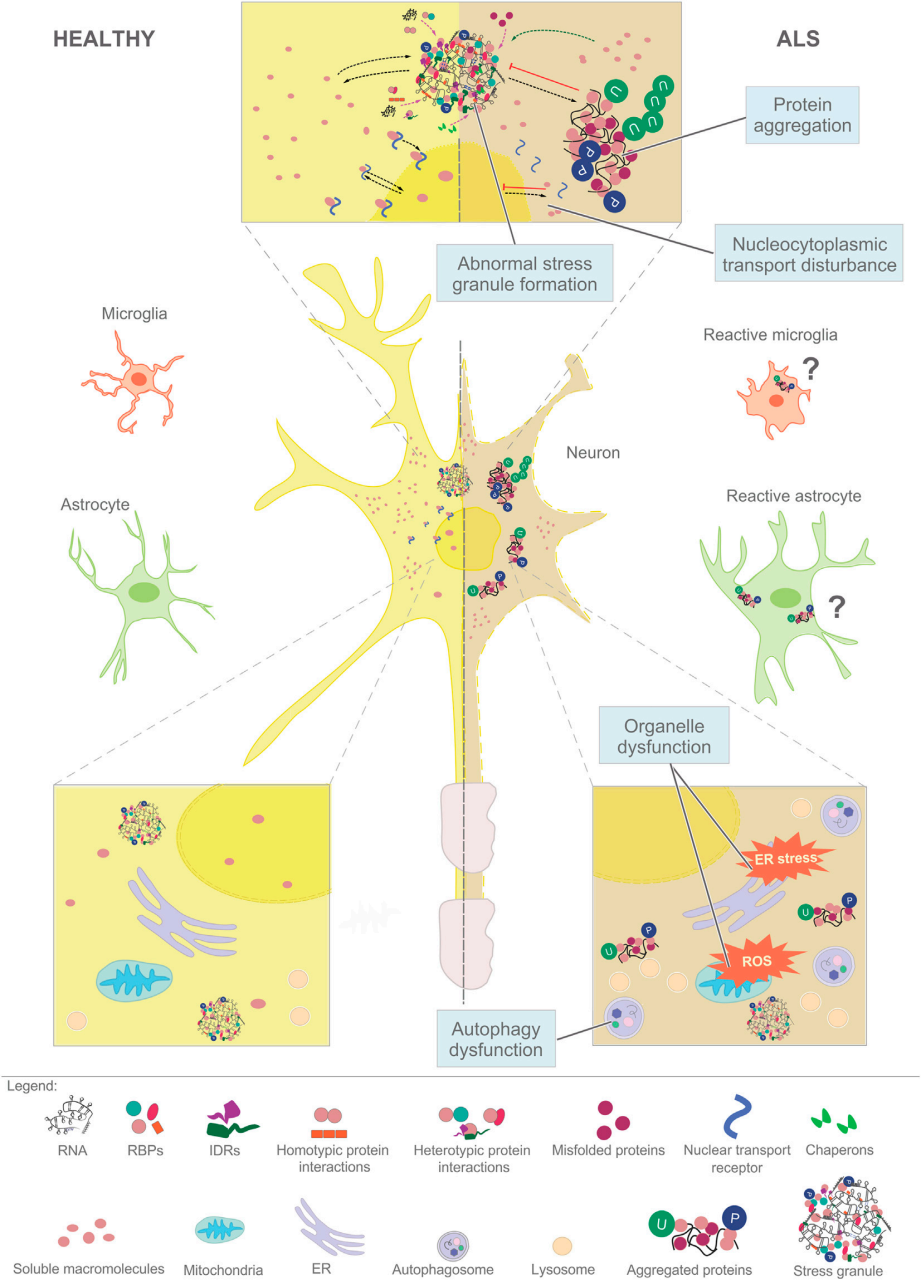
Aberrant phase separation of RNA-binding proteins (RBPs) plays a central role in the cellular pathology of ALS. Macromolecules such as RNAs and proteins assemble in dynamic and reversible condensates in healthy neurons and glia (left), that mature into stress granules (top, center) and insoluble aggregates destined for degradation (right). The cellular pathways that affect the LLPS of RNPs include (i) nucleocytoplasmic transport, (ii) assembly of stress granules, (iii) aggregation driven by the accumulation of misfolded proteins, (iv) organellar dysfunction that leads to ROS and persistent stress, and (v) failed autophagy-lysosome pathway. Similar pathways might play a role in glial cells.

In this review, we outline current knowledge on the ALS-related protein condensations in neurons and glia. In particular, we emphasize how disrupted LLPS of RNA-binding proteins lead to their aggregation. For the roles of two well-studied RBPs, TDP-43 and FUS in frontotemporal dementia, we refer to the review by Carey and Guo within this Special Issue. The aberrant RBP condensates can even trap folded proteins which do not undergo LLPS such as SOD1 (Mateju et al., 2017; Pakravan et al., 2021) and alter the intracellular organelle trafficking (Ling et al., 2019; Trnka et al., 2021). Understanding the mechanisms how LLPS of several ALS-associated RBPs corroborates with the intracellular organelle trafficking (Figure 1) opens novel directions for disease treatment.

## Protein Aggregates are Clinical Hallmark in ALS

A common feature of both sALS and fALS is the presence of protein aggregates in the cytosol of neurons and glia. Early studies reported aggregates that are ubiquitin-positive dense, irregular or filamentous (skein-like) inclusions in the spinal cord motoneurons in fALS and sALS patients (Leigh et al., 1991). DNA/RNA-binding proteins, TDP-43 and FUS are normally localized in the nucleus where they act as RNA metabolism regulators (Andersson et al., 2008; Buratti and Baralle, 2008). In ALS, TDP-43 and FUS mislocalize from the nucleus to the cytoplasm where they aggregate and appear in ALS-related inclusions (Blokhuis et al., 2013). Within aggregates, TDP-43 is frequently post-translationally modified by ubiquitination and/or phosphorylation (Neumann et al., 2006, 2009; Mackenzie et al., 2007; Hasegawa et al., 2008; Bodansky et al., 2010; Brettschneider et al., 2013), aberrant cleavage (Neumann et al., 2006; Altman et al., 2021) and protein misfolding (Prasad et al., 2019). On the other hand, both mutated and wild-type SOD1 are found in the cytosolic aggregates in the spinal cord and cortical motoneurons of fALS patients bearing SOD1 mutation (Jonsson et al., 2004, 2008). Protein aggregates are also identified in glial cells surrounding degenerating motor neurons. SOD1 aggregates are present in astrocytes and microglia (Jonsson et al., 2004; Stamenković et al., 2017) and are related to a change in cell morphology (Stamenković et al., 2017). Similarly, TDP-43 inclusions are found in the glial cells of both gray and white matter spinal cord, in sALS and some fALS patients (Arai et al., 2006; Mackenzie et al., 2007; Brettschneider et al., 2013) suggesting the important role of glial cells in ALS pathology (Vahsen et al., 2021).

Protein aggregates in ALS are heterogenous. For example, SOD1 is occasionally found in the aggregates in motoneurons of ALS patients with C9orf72 and FUS mutations (Forsberg et al., 2019). TDP-43-positive inclusions were identified in fALS patients with C9orf72 expansion (Collins et al., 2012), but not in patients bearing FUS or SOD1 mutations (Mackenzie et al., 2007; Tan et al., 2007; Vance et al., 2009) indicating distinct pathways of aggregate formation. However, studies in SOD1^A4T^ patients point to the interaction of SOD1 and TDP-43 as confirmed by their co-immunoprecipitation (Volkening et al., 2009). The data on ALS patients only provide the histopathological picture at the terminal stage of the disease. Nonetheless, induced pluripotent stem cells (iPSCs) derived from patient fibroblasts recapitulate protein pathology successfully (Mattis and Svendsen, 2011; Burkhardt et al., 2013; Fujimori et al., 2018). iPSC-derived motoneurons from patients carrying FUS or TDP-43 mutations show mislocalization of mutated proteins into the cytoplasm, their phosphorylation (Fujimori et al., 2018), and aggregate formation (Bilican et al., 2012; Egawa et al., 2012; Fujimori et al., 2018). iPSC-derived neurons from patients with sALS display a presence of cytosolic and intranuclear hyperphosphorylated TDP-43 aggregates similar to those in sALS *post mortem* tissues (Burkhardt et al., 2013; Fujimori et al., 2018). Astrocytes generated from iPSCs harboring TDP-43 mutation show elevated levels of cytoplasmic TDP-43 and increased astrocytic cell death (Serio et al., 2013).

Several mechanisms have been proposed to underlie ALS pathology, including oxidative stress, mitochondrial dysfunction, stress at the endoplasmic reticulum (ER), disruption of RNA metabolism, neuroinflammation, glutamate excitotoxicity (rev. in Taylor et al., 2016; Mejzini et al., 2019). They are all linked to the presence of intracellular protein aggregates, which are often composed of RNA-binding proteins, able to undergo liquid-liquid phase separation (LLPS) (Molliex et al., 2015; Patel et al., 2015) (Figure 1).

## Liquid-Liquid Phase Separation: Mechanism for Assembly of RNP Granules

LLPS is a thermodynamic process in which two (or more) components demix from a homogenous solution to form two or more distinct phases. In the context of cell biology, LLPS is a mechanism where biomolecules demix from a crowded cytosol or nucleoplasm to form distinct compartments referred to as biomolecular condensates. The critical features of biomolecular condensation include multivalent, low-affinity interactions of biomolecules present at high local concentrations. Thus far, RNA-RNA, RNA-protein and protein-protein interactions (Banani et al., 2017; Mittag and Parker, 2018; Van Treeck et al., 2018) are all shown to phase separate. Moreover, the entire pools of membrane-bound organelles can actively assemble in mesoscale compartments through LLPS such as clusters of synaptic vesicles (Milovanovic et al., 2018; Hoffmann et al., 2021) and stacks of Golgi apparatus (Rebane et al., 2020; Ziltener et al., 2020).

LLPS emerged as a mechanism for dynamic and reversible assembly of ribonucleoprotein (RNP) complexes (Han et al., 2012; Brangwynne, 2013; Lin et al., 2015; Molliex et al., 2015; Banani et al., 2017; Mittag and Parker, 2018). Number of functionally distinct RNP granules is suggested to undergo LLPS, including cytoplasmic RNP granules, processing (P)-bodies and stress granules (SGs)—sites of translationally silenced mRNAs and/or RNA degradation (Decker and Parker, 2012), neuronal granules—crucial for mRNA packing, translational control, and axonal transport (Fernandopulle et al., 2021).

The intrinsically disordered regions (IDRs)—amino acid sequences that do not fold in any specific secondary or tertiary structure—play a central role in RNP granule assembly and dynamics. For example, the missense mutation in TIA-1 IDR inhibits RNP granule disassembly (Mackenzie et al., 2017) and promotes the accumulation of non-dynamic RNP granules enriched in insoluble TDP-43 (Mackenzie et al., 2017). RNP granule formation can be facilitated by IDR interactions via local structures such as the α-helix of C-terminal domain of TDP-43 essential for its LLPS (Conicella et al., 2016, 2020) which has the propensity to self-associate. Also, IDR of FUS forms heterotypic interactions with GFP-fused IDR either of FUS or other proteins such as hnRNPA1 (Kato et al., 2012). Moreover, ALS linked mutations are very often located in the IDRs of LLPS related proteins (for details, see Table 1) altering their LLPS propensity and/or RNP granule dynamics.

**TABLE 1:** An overview of ALS-linked mutations in LLPS-related proteins.

Protein	Mutations implicated in ASL	Mutation region	Source in addition to Uniprot
ANXA11			
(*Annexin A11*)	P31R, P36R, G38R^#•^, D40G^#•^, A58_Q187del, G89S, Q97X, V128M, G137R, G175R^•^, G189E	LCD; NTD	Smith et al. (2017); Tsai et al. (2018); Zhang et al. (2018); Nahm et al., 2020
	V208L, G228Lfs29, S229R, R235Q^#^, A293V,R302C, I307M, T321N, R346C, Q362L, A367V, L383_V392del, H390P, R456H^•^, I457V, G491R	ANX homology domains (1-4)	Smith et al. (2017); Tsai et al. (2018); Zhang et al. (2018); Liu et al. (2019); Nahm et al. (2020)
ATXN2			
(*Ataxin-2*)	polyQ repeats of different length	PolyQ region within NTD 166-187	Chiò et al. (2015); Blokhuis et al. (2016); Sproviero et al. (2017)
hnRNPA1			
(*Heterogeneous nuclear ribonucleoprotein A1*)	D262V^#•^	Prion-like domain; CTD	Molliex et al. (2015); Liu et al. (2016); Beijer et al. (2021)
	D262N^#^, Q277K, G283R	LCD; CTD	Kim et al. (2013); Gilpin et al. (2015); Liu et al. (2016)
	P288S, P288A	LCD; NLS; CTD	Liu et al. (2016); Naruse et al. (2018)
	G304Nfs^#^	LCD; CTD	Beijer et al. (2021)
	(321Ext)6^#^, (321Qext)6^#^	Extension on CTD	Beijer et al. (2021)
hnRNPA2/B1			
*(Heterogeneous nuclear ribonucleoprotein A2/B1)*	D290V	LCD; CTD	Kim et al. (2013)
FUS			
(*Fused in sarcoma*)	S57del, G144E^#^, G154E^#^, G156D^#^, G156E^#^, G187S^#^, G191S, R216C, G225V^#^, G230C^#^, R234C, R234L, R244C^#^, M254V	Prion-like domain; NTD	Belzil et al. (2009); Kwiatkowski et al. (2009); Ticozzi et al. (2009); Corrado et al. (2010); Rademakers et al. (2010); Merner et al. (2012); Nomura et al. (2014); Patel et al. (2015); Niaki et al. (2020)
	S402_P411delinsGGGG	RGG1 motif	DeJesus-Hernandez et al. (2010)
	S462F, G466VfsX14	RGG2 motif	DeJesus-Hernandez et al. (2010); Groen et al. (2010)
	R495X, G507D, K510E, S513P, R514G, R514S, G515C, H517Q, H517P, R518G, R518K, R521G, R521H, R521C, R552G, R524S, R524W, R524T, P525L	NLS; CTD	Kwiatkowski et al. (2009); Corrado et al. (2010); Dormann et al. (2010); Yan et al. (2010); Gal et al. (2011); Niu et al. (2012); Nomura et al. (2014)
TAF15			
(*TATA-box binding protein associated factor 15*)	A31T	Prion-like domain; NTD	Ticozzi et al. (2011b)
	M368T, D386N, R388H, G391E^#^, R395Q, R408C^#^, G452E, G464_G471del, G473E, R474_D481del	LCD; CTD	Couthouis et al. (2011); Ticozzi et al. (2011b)
TDP-43			
(*TAR DNA binding protein 43*)	A90V	NLS; NTD	Winton et al. (2008); Chiang et al. (2012)
	D169G^#^	RRM1	Kabashi et al. (2008); Nonaka et al. (2009); Austin et al. (2014)
	K263E^#^, N267S	RRM2	Kabashi et al. (2008); Corrado et al. (2009); Austin et al. (2014)
	G287S, G290A, S292N, G294A, G294V, G295R, G295S, G298S, M311V, A315T, A321V, A321G, Q331K^#^, S332N, G335D, M337V^#^, Q343R^#^, N345K^#^, G348C, G348V,N352T, N352S, R361S^#^, P363A, Y374X, N378D, S379P, S379C, A382T, A382P, I383V, G384R, N390D^#^, N390S, S393L	Prion-like domain; CTD	Sreedharan et al. (2008); Van Deerlin et al. (2008); Gitcho et al. (2008); Kabashi et al. (2008); Bäumer et al. (2009); Lemmens et al. (2009); Corrado et al. (2009); Daoud et al. (2009); Johnson et al. (2009); Kamada et al. (2009); Ling et al. (2010); Barmada and Finkbeiner, (2010); Xiong et al. (2010); Elden et al. (2010); Kirby et al. (2010); Ticozzi et al. (2011a); Chiang et al. (2012); Lattante et al. (2012); Zou et al. (2012)
TIA-1			
(*T-cell-restricted intracellular antigen-1*)	P362L^#•^, A381T^#•^, E384K^#•^	LCD	Hackman et al. (2013); Mackenzie et al. (2017)

CTD, C-terminal domain; NTD, N-terminal domain; LCD, low-complexity domain; RRM, RNA recognition motif; NLS, nuclear localization signal; RGG, arginine-glycine-glycine; PolyQ, polyglutamine.

#LLPS propensity and/or aggregation affected; SG dynamics affected.

Reversibility is a key feature of biomolecular condensates such as RNPs, with post-translational modifications playing a key role in regulating this process (Kato et al., 2012; Monahan et al., 2017; Wang et al., 2018; Hofweber and Dormann, 2019; Schisa and Elaswad, 2021). Phosphorylation of GFP-tagged IDR of FUS impairs its retention by preformed FUS hydrogels (Han et al., 2012). Mutations in IDR that increase number of FUS phosphorylation sites impair polymerization propensity and SG recruitment (Kato et al., 2012). On the other hand, phospho-double mutants within the FUS IDR core disrupt droplet disassembly (Murray et al., 2017). Therefore, a tight balance of (de)phosphorylation determines RNP granule clearance and maintenance of their fluid state. Beyond phosphorylation, post-translational modifications play a central role in the regulation of RNA-binding proteins implicated in ALS (Hofweber and Dormann, 2019; Sternburg et al., 2021).

## ALS-Related Mutations Disrupt Phase Separation of RNA Granules Resulting in the Formation of Insoluble Aggregates

### Nucleic Acid-Binding Proteins, FUS and TDP-43, Play a Critical Role in the Pathology of ALS

Several fALS are associated with the missense mutations in TDP-43 (Kwon et al., 2012) and FUS (Deng et al., 2014). Interestingly, ALS-related mutations in the α-helix of C-terminal disordered domain within TDP-43 disrupt LLPS of TDP-43 resulting in the formation of aggregates (Conicella et al., 2016). Moreover, TDP-43 mutant RNP granules in axons of rat primary cortical neurons display increased viscosity and impaired transport dynamics compared to the wild-type RNP granules (Gopal et al., 2017). Similarly, optogenetic enhancement of TDP-43 oligomerization accelerates ALS-related pathologies in the spinal motor neurons (Asakawa et al., 2020).

Another ALS-linked protein, FUS, has been reported to shuttle between nucleus and cytoplasm in a highly dynamic manner (Patel et al., 2015). In the nucleus, FUS enriches at the transcriptional sites or sites of DNA damage, while upon heat shock, it relocates to SGs in the cytoplasm (Bosco et al., 2010; Rulten et al., 2014; Patel et al., 2015). This relocation of FUS is affected in ALS patients where FUS aggregates in cytoplasmic inclusions (Dormann et al., 2010). FUS undergoes LLPS (Kato et al., 2012; Patel et al., 2015) and this process is abolished in FUS mutant lacking N-terminal IDR (Patel et al., 2015). ALS-linked mutations in the IDR of FUS (e.g., G156E) promote aggregate formation (Nomura et al., 2014) through molecular aging of FUS condensates that accelerate the conversion of FUS liquid droplets to fibrillar aggregates (Patel et al., 2015). Similarly, ALS-related mutations in hnRNPA1 promote fibrilization and seeding of hnRNPA1 fibrils within the fluid droplet (Molliex et al., 2015). Altogether, these studies strongly suggest that the aberrant phase transition of RNP granules is involved in the onset of ALS.

### RNA Buffers Phase Separation Behavior of ALS Linked RBPs

In recent years, RNA molecules are emerging as central regulators of RNP granule formation and dynamics (rev. in Van Treeck et al., 2018; Tian et al., 2020) and can undergo phase separation (Jain and Vale, 2017). RNA molecules are critical for regulating the phase behavior of ALS-linked proteins, including FUS, TDP-43 and hnRNPA1 (Banerjee et al., 2018; Maharana et al., 2018; Niaki et al., 2020). Specifically, the ratio of RNA to protein has an important role in promoting RNA droplet formation: at a low RNA/protein ratio, RNP droplets are prompted to form, while high RNA/protein ratios lead to droplet dissolution (Elbaum-Garfinkle et al., 2015; Maharana et al., 2018). Moreover, long RNAs can act as scaffolds that further promote FUS droplet formation (Maharana et al., 2018). This implicates the importance of both RNA concentration and intrinsic features in the regulation of ALS-linked RNP granules assembly (Zhang et al., 2015; Sanchez de Groot et al., 2019). The formation of ALS-linked FUS mutant aggregates can be prevented by addition of RNA molecules *in vitro* (Maharana et al., 2018)*.* In addition, ALS-linked mutations in the C-terminal domain of FUS alter RNA binding and promote formation of aggregated FUS-containing RNP complexes (Niaki et al., 2020). Altogether, these findings indicate that specific RNA sequences could prevent the aberrant aggregation of ALS-linked RBPs (Banerjee et al., 2018; Maharana et al., 2018; Niaki et al., 2020).

### ALS Mutations Disrupt Nuclear-Cytoplasmic Shuttle

Disbalance between nuclear and cytoplasmic transport of ALS-linked proteins could contribute to their toxic accumulation in cytoplasmic inclusions of ALS/FTD patients. Indeed, ALS-associated mutations in nuclear localization sequences (NLS) of FUS are responsible for the disruption of FUS localization in the nucleus (Dormann et al., 2010). The FUS NLS interacts with nuclear transport receptor (NTR) Karyopherin-β2 (Kapβ2) (Lee et al., 2006; Dormann et al., 2012). This interaction drives FUS nuclear import (Dormann et al., 2010) and is critical for phase separation of FUS (Hofweber et al., 2018; Yoshizawa et al., 2018). In fact, Kapβ2 also dissolve phase-separated hnRNPA1 and FUS (Guo et al., 2018). Kapβ2 specifically blocks FUS phase separation *in vitro* (Hofweber et al., 2018; Yoshizawa et al., 2018).

Chaperoning of FUS by Kapβ2 is mediated via RGG domain (Dormann et al., 2012), crucial for LLPS of RNA-binding proteins (Hofweber et al., 2018; Schuster et al., 2020; Wang et al., 2021). All RGG repeats of FUS undergo post-translational methylation by PRMT1 or PRMT8 (Scaramuzzino et al., 2013). Interestingly, it has been observed that in patients, arginine methylation of FUS is disrupted (Dormann et al., 2012; Suárez-Calvet et al., 2016) leading to the impairment of FUS LLPS and localization within SGs (Yoshizawa et al., 2018) implicating the interplay between post-translational modifications and nuclear cytoplasmic shuttle in regulating RNP distribution to different cellular compartments. Nuclear transport disruption have also been implicated in TDP-43 pathology (Chou et al., 2018). Interestingly, TDP-43 fibrils are not engaged by Kapβ2 but by Impα and Kapβ1 via its NLS. In addition, it has been shown that R-rich dipeptide repeats similar to those found in *C9orf72* hexanucleotide repeats, can bind NTRs such as Impα, Impβ, Kapβ2 (Hutten et al., 2020) competing with NLS-containing cargos such as TDP-43. Beyond being just a signal that promotes nuclear import, NLS as well as the availability of NTRs play an anti-aggregation role, ensuring that the nuclear cargo is chaperoned and non-aggregated in the cytoplasm.

## Stress Granule Assembly and Dynamics are Altered in ALS

Upon exposure to stress, a pool of mRNAs recruit a set of specific RBPs, such as G3BP1 (Kedersha et al., 2013, 2016; Jain et al., 2016) and nucleate a special type of RNP granules, so-called stress granules (SGs). SGs are membraneless organelles (Kedersha et al., 2005; Baradaran-Heravi et al., 2020; Malik and Wiedau, 2020) that allow the prompt response of the cell to stress by regulating protein synthesis (Ashe et al., 2000; Besse and Ephrussi, 2008; Spriggs et al., 2010). Upon stress relief SGs disassemble allowing re-initiation of mRNA translation (Buchan and Parker, 2009; Decker and Parker, 2012; Panas et al., 2016; Baradaran-Heravi et al., 2020). SGs can also act as sites of mRNA triage, where transcripts are routed to either translation, degradation or packing (Hofmann et al., 2021).

In CNS, SG dynamics is cell-type dependent as their distribution in neurons is perinuclear while in astrocytes SGs are localized toward the cell periphery (Khalfallah et al., 2018). Nonetheless, TDP-43 is required for maintaining SG dynamics in both cell types (Aulas et al., 2012). Misregulation of SG dynamics is linked to aggregation in ALS (Li et al., 2013). Many ALS-linked proteins, including FUS, TDP-43, TIA-1, hnRNPA1, Ataxin-2, C9orf72 repeats have all been shown to localize in SGs (Nonhoff et al., 2007; Bentmann et al., 2012; Dewey et al., 2012; Li et al., 2013; Ramaswami et al., 2013; Boeynaems et al., 2017). In fact, mutations of these proteins affect the LLPS of SGs. For example dipeptides derived from *C9orf72* hexanucleotide repeats not only undergo LLPS themselves (Boeynaems et al., 2017; White et al., 2019), but also affect SG dynamics in cells (Boeynaems et al., 2017).

It has been shown that molecular chaperones play an important role in preventing the assembly of aberrant SGs (Ganassi et al., 2016; Mateju et al., 2017; Lu et al., 2021; Yu et al., 2021). HSP27 and HSP70 were shown to localize in SGs containing misfolded proteins (Mateju et al., 2017). Furthermore, chemical inhibition of HSP70 increased the population of SGs containing misfolded proteins, including mutated SOD1 (Mateju et al., 2017). Moreover, HSP70 was shown to be crucial for maintaining the fluidity of anisosome-nuclear inclusions (Ganassi et al., 2016) and disassembly of TDP-43 cytoplasmic droplets (Lu et al., 2021). Reduced levels of HSP27 are reported in motor neurons of patients with TDP-43-associated ALS (Lu et al., 2021). Although SOD1 as a folded protein does not undergo LLPS, it has been shown that mutant SOD1 (mSOD1) has a tendency to accumulate in SGs in ALS (Mateju et al., 2017). Unlike wild-type, mutant SOD1 is identified in G3BP1- and TIA-1-positive SGs in the spinal cord tissue from SOD1^G93A^ mice and SOD1 ALS patients (Gal et al., 2016; Mateju et al., 2017; Lee et al., 2020) and contributes to the impaired dynamics of SGs (Lee et al., 2020). mSOD1 directly interacts with the RNA-binding domain of G3BP1 and affects SG dynamics as the presence of mSOD1 delayed the formation of G3BP1-positive SGs in response to osmotic stress (Gal et al., 2016). Moreover, mSOD1-containing SGs tend to increase ER stress (Rajpurohit et al., 2020). Similarly, mSOD1 interacts with TIA-1 containing SGs leading to their impaired dynamics (Lee et al., 2020).

Another important mechanism in regulating the aberrant SGs is autophagy (Ganassi et al., 2016; Mateju et al., 2017). If persistent upon removal of stress, SGs undergo the process of autophagy (Buchan et al., 2013). ALS-associated misfolded proteins are targeted to aggresomes (Kawaguchi et al., 2003) subsequently degraded by autophagy (Mateju et al., 2017). The aberrant phase separation of FUS leads to the formation of cytosolic aggregates in a concentration-dependent fashion and lysosomes are juxtaposed to these aggregates (Trnka et al., 2021). In line with these findings, increased lysosomal activity and enhanced autophagy are also reported in astrocytes derived from ALS patients (Rajpurohit et al., 2020). Recently, increased recruitment of small ubiquitin-like modifier (SUMO) ligases into the SGs is observed upon stress exposure leading to SUMOylation of proteins necessary for SG disassembly (Marmor-Kollet et al., 2020), and its disruption occurs in ALS patients.

## Open Questions and Future Directions

Intensive research has been focused on cellular mechanisms underlying ALS. Here we summarized the emerging roles of LLPS in cellular pathology of ALS. In particular, we highlighted TDP-43 and FUS, two proteins enriched in RNP granules. Finally, we discuss how the regulation of RNP dynamics is critical for the reversible assembly and clearance of SGs, which if not regulated, lead to aberrant phase separation and aggregation. Together, these studies open several important questions. For example, apart from neurons, emerging evidence suggests that glial cells play an important role in the ALS onset and progression (Vahsen et al., 2021). However, whether the cytosolic-nuclear dynamics of TDP-43 or the SG dynamics differ between neurons and glia remains unclear.

Another important aspect is the use of animal models of ALS (Picher-Martel et al., 2016; Morrice et al., 2018) that are a valuable tool to improve the selection of novel therapeutic candidates. However, many drugs that showed an efficient effect in rodents failed later in clinical trials (Moujalled and White, 2016). To date, only a few drugs are officially approved for ALS treatment (Liscic et al., 2020), which all show limited therapeutic benefit for ALS patients (Brown and Al-Chalabi, 2017), emphasizing the need for defining novel targets.

The nuclear-cytosolic shuttle promotes the development of new targets. For example, a TDP-43 A90V mutation within its NLS leads to accumulation of insoluble TDP-43 (Winton et al., 2008). Interestingly, TDP-43 NLS is a target of TDP-43 PARylation which promotes its liquid demixing (McGurk et al., 2018). These new targets need to take into account both the neurons and glia, and account for LLPS-derived mechanisms that might result in effects visible only when the backup surveillance systems fail, as in aging or upon acute stress.

Recent findings demonstrate the association of SGs with several membrane-linked compartments such as ER, lysosomes and mitochondria (Liao et al., 2019; Lee et al., 2020; Amen and Kaganovich, 2021; Trnka et al., 2021) raising the interest in SG physiological properties and their implication in ER and oxidative stress in ALS. These are precisely the mechanisms targeted by the most recent drug AMX0035 (tauroursodeoxycholic acid and sodium phenylbutyrate), which is at the late stages of approval by FDA for clinical use.

Finally, the aberrant phase-separation of RNA-binding proteins is just one side of the coin. To understand the cellular response to aggregates, intense research needs to focus on the organelles, particularly lysosomes and autophagy system. Many fALS cases are associated with genes involved in the function of lysosomes, and impaired lysosome trafficking has been reported in ALS post-mortem tissue, as reviewed in Root et al. (2021). Hence, a cell-specific view that will focus both on the protein localization and the changes in concentrations, as well as the intracellular trafficking, promises to give necessary insights into the cellular pathophysiology of ALS.
